# PrP Expression, PrP^Sc^ Accumulation and Innervation of Splenic Compartments in Sheep Experimentally Infected with Scrapie

**DOI:** 10.1371/journal.pone.0006885

**Published:** 2009-09-03

**Authors:** Randi Sørby, Lars Austbø, Charles McL. Press, Grethe Skretting, Thor Landsverk, Arild Espenes

**Affiliations:** 1 Department of Basic Sciences and Aquatic Medicine, Norwegian School of Veterinary Science, Oslo, Norway; 2 Haematological Research Laboratory, Ullevål University Hospital, Oslo, Norway; University of Liverpool, United Kingdom

## Abstract

**Background:**

In prion disease, the peripheral expression of PrP^C^ is necessary for the transfer of infectivity to the central nervous system. The spleen is involved in neuroinvasion and neural dissemination in prion diseases but the nature of this involvement is not known. The present study undertook the investigation of the spatial relationship between sites of PrP^Sc^ accumulation, localisation of nerve fibres and PrP^C^ expression in the tissue compartments of the spleen of scrapie-inoculated and control sheep.

**Methodology/Principal Findings:**

Laser microdissection and quantitative PCR were used to determine PrP mRNA levels and results were compared with immunohistochemical protocols to distinguish PrP^C^ and PrP^Sc^ in tissue compartments of the spleen. In sheep experimentally infected with scrapie, the major sites of accumulation of PrP^Sc^ in the spleen, namely the lymphoid nodules and the marginal zone, expressed low levels of PrP mRNA. Double immunohistochemical labelling for PrP^Sc^ and the pan-nerve fibre marker, PGP, was used to evaluate the density of innervation of splenic tissue compartments and the intimacy of association between PrP^Sc^ and nerves. Some nerve fibres were observed to accompany blood vessels into the PrP^Sc^-laden germinal centres. However, the close association between nerves and PrP^Sc^ was most apparent in the marginal zone. Other sites of close association were adjacent to the wall of the central artery of PALS and the outer rim of germinal centres.

**Conclusions/Significance:**

The findings suggest that the degree of PrP^Sc^ accumulation does not depend on the expression level of PrP^C^. Though several splenic compartments may contribute to neuroinvasion, the marginal zone may play a central role in being the compartment with most apparent association between nerves and PrP^Sc^.

## Introduction

Scrapie in sheep is one of a group of fatal neurodegenerative disorders called prion diseases or transmissible spongiform encephalopathies (TSEs). A conformational transformation from an α-helix rich normal prion protein (PrP^C^) to a β-sheet rich, insoluble and partially protease resistant, disease-associated form of the protein (PrP^Sc^) results in the accumulation of the abnormally folded protein mainly in lymphatic tissues and the nervous system [1]. The gastrointestinal tract is the most likely route of entry of the infectious agent in natural disease [2], [3] and a lymphoreticular phase with PrP^Sc^ accumulation in lymphoid nodules of the gut-associated lymphatic tissues and other peripheral lymphatic tissues usually precedes the spread of the infection to the central nervous system [4], [5]. The peripheral expression of PrP^C^ is essential for transfer of infectivity to the central nervous system [6] and the efficiency of neuroinvasion is influenced by the level of expression of PrP^C^ [7].

Using a combination of laser microdissection and quantitative PCR, Austbø *et al*. [8] found that the expression of PrP mRNA varied between tissue compartments in the ileal Peyer's patch of sheep, with highest levels of expression in the nerve rich compartments of the outer submucosa and muscle layers. The lowest level of PrP mRNA expression was found in the submucosal lymphoid nodules of the Peyer's patch, which is the tissue compartment that houses the major accumulation of PrP^Sc^ in diseased animals. The investigation of Peyer's patch nodules in sheep experimentally infected with scrapie showed that while PrP mRNA levels were significantly above the levels in unaffected control animals, the overall PrP mRNA expression in the nodule compartment remained low compared with other tissue compartments in the gut wall of the ileal Peyer's patch [9]. These findings suggested that high tissue expression of PrP^C^ was not essential at the site of accumulation of PrP^Sc^. However, Austbø et al. [9] did not investigate the compartmentalisation of PrP^C^ expression in other peripheral lymphatic tissues, nor did they investigate the PrP^C^ expression and PrP^Sc^ accumulation in relation to possible sites of neuroinvasion.

The role of the spleen in neuroinvasion and neural dissemination of prion diseases has been extensively investigated. Studies in mice [10] and hamsters [11] have shown that the scrapie agent can spread to the spinal cord via the splanchnic nerves of the spleen. The spleen is richly innervated by sympathetic noradrenergic nerve fibres and sympathectomy or sympathetic hyperinnervation of the spleen has been shown to delay or hasten respectively the development of scrapie in mice [12]. While the accumulation of PrP^Sc^ in the germinal centres of peripheral lymphatic tissues is well documented [13], it is also known that only a few delicate nerve fibres extend into germinal centres [14]. Studies in the sheep spleen have shown that tissue compartments other than lymphoid nodules can contain significant accumulations of PrP^Sc^. In sheep naturally or experimentally exposed to the scrapie agent, Heggebø *et al.* [3], [15] demonstrated a noticeable accumulation of PrP^Sc^ in the marginal zone of the spleen. Anatomical investigations of the mammalian spleen have demonstrated that the marginal zone and other splenic compartments such as the periarterial lymphatic sheaths (PALS) are richly innervated and contain numerous nerve fibres [16]–[18]. It would be expected that the contribution of an anatomical compartment to neuroinvasion by the scrapie agent would be influenced by the level of PrP^C^ expression, the level of PrP^Sc^ accumulation and the degree of innervation.

The present paper addresses the spatial relationship between sites of PrP^Sc^ accumulation, localisation of nerve fibres and PrP^C^ expression in the tissue compartments of the spleen of scrapie-inoculated and control sheep. A range of techniques is used including laser microdissection and quantitative PCR to determine PrP mRNA expression in tissue compartments as well as immunohistochemical protocols to distinguish PrP^C^ and PrP^Sc^. We show that tissue compartments with low expression of PrP mRNA harbour accumulations of PrP^Sc^. The close association of nerves and PrP^Sc^ was most apparent in the marginal zone, but was also evident immediately adjacent to the central artery of the PALS and abutting the outer rim of lymphoid nodules. Some nerves were observed to accompany blood vessels supplying the PrP^Sc^-laden germinal centres.

## Materials and Methods

### Animals

All animals were handled in strict accordance with good animal practice as defined by the Norwegian National Animal Research Authorities, and all animal work was approved by the Norwegian National Animal Research Authorities. Ten sheep of the Norwegian white breed with the susceptible PrP genotype V_136_R_154_Q_171_/V_136_R_154_Q_171_ (VRQ/VRQ) were included in the study. Five lambs were inoculated with a single dose of a 30% (w/v) homogenate of brain tissue (5 g brain tissue) by stomach tube at the age of 6–8 weeks. The homogenate contained frozen brain tissue from confirmed scrapie cases with the VRQ/VRQ genotype. Five age matched sheep served as controls. The animals were kept in separate isolation facilities and euthanised 10–18 months post inoculation. Frozen tissue from the cerebellum of a normal healthy one month old lamb was collected for evaluation of immunohistochemical and *in situ* hybridisation (ISH) protocols.

### Laser microdissection

Frozen spleen sections (14 µm) were placed on special membrane slides for laser microdissection (Molecular Machines and Industries). The sections were air-dried at room temperature and stored at −80°C until use. The slides were stained with RNase-free haematoxylin and air dried before microdissection using a SLµCut laser microdissection system (Molecular Machines and Industries). This system is equipped with an automated UV laser beam dissection system coupled to video imaging enabling dissection of separate splenic compartments. To preclude internal variation, several pieces of each desired compartment were microdissected to obtain an area corresponding to 1×10^6^ µm^2^, i.e. at least 10 different lymphoid nodules from each animal. The microdissected areas of lymphoid nodules, marginal zone, periarterial lymphatic sheath, red pulp and trabeculae were collected in separate tubes for PrP mRNA analysis.

### RNA extraction

RNA from laser-captured tissue was isolated using the Absolutely RNA Nanoprep kit (Stratagene). The manufacturer's protocol was followed including the optional DNase step. The RNA was eluted into 20 µl elution buffer, and stored at –80°C.

### Quantitative Real-Time RT-PCR

Quantitative real-time RT-PCR was performed using one-step qPCR core kit (Eurogentec). Primers were designed to span across intron sections using PrimerExpress 1.5 (Applied Biosystems). The expression level was measured with relative quantification using glyceraldehyde-3-phosphate dehydrogenase (GAPDH) as the reference gene. Each quantification target was amplified in triplicate samples and a control lacking the template for each master mix was always included in the experiments.

Primers and hybridisation probes used for the quantitative real-time RT-PCR were as follows: *Ovis aries* PrP: forward 5′-TCCCAGAGACACAGATCCAACTT-3′, reverse 5′-GATCCAACTGCCTATGTGGCTT-3′, probe 5′-FAM-ACCATGATGACTTCTATCTGCTGTGATTCAGCT-TAMRA-3′. *Ovis aries* GAPDH: forward 5′-TGATTCCACCCATGGCAAGT-3′, reverse 5′-CCACGTACTCAGCACCAGCAT-3′, probe 5′-FAM-TCCACGGCACAGTCAAGGCAGAGAA-TAMRA-3′. Real-time RT-PCR was carried out in an ABI PRISM 7700 (Applied Biosystems) using the following uniform temperature profile: 30 min at 48°C (reverse transcription), then 10 min at 95°C (denaturation), followed by 40 cycles of 30 s at 95°C, 15 s at 56°C and 60 s at 60°C. The same cycling profile was used for all real-time RT-PCRs. The data was analysed using Sequence Detection System v1.9.1 (Applied Biosystems).

### PrP^C^ and PrP^Sc^ immunohistochemistry

For detection of PrP^C^ and PrP^Sc^, the immunohistochemical procedure was identical but the pretreatment steps differed. For PrP^C^ detection, 7 µm thick cryosections were fixed in fresh 0.5% paraformaldehyde/0.25% glutaraldehyde in phosphate-buffered saline (PBS), pH 7.4 for 15 min and autoclaved at 121°C for 4 minutes in 0.1 M citrate buffer pH 6.0. For PrP^Sc^ detection, formalin fixed tissue blocks were immersed in 98% formic acid for 1 hour prior to paraffin embedding, and slides were autoclaved at 121°C for 5 minutes in 0.1 M citrate buffer pH 6.0 and treated with trypsin (Difco) at 37°C for 5 minutes.

The rest of the immunohistochemical procedure was identical for both the PrP^C^ and PrP^Sc^ protocol. To inhibit endogenous peroxidase, the tissue sections were treated with 3% H_2_O_2_ in methanol for 20 minutes. The sections were further incubated in a blocking solution, which consisted of avidin (Cat. no. SP-2001, Vector Laboratories) diluted 1∶6 in TNB blocking buffer (0.1 M Tris-HCl, pH 7.5, 0.15 M NaCl, 0.5% blocking reagent supplied in the TSA kit). Immunolabelling was performed using a tyramide signal amplification system, the indirect TSA-kit (NEN^TM^ Life Science Product) according to the manufacturer's protocol. The tissue sections were incubated at 4°C overnight with the mouse monoclonal antibody L42 (kindly provided by Dr. Martin H. Groschup) diluted 1∶150 and 1∶300 for the PrP^C^ and PrP^Sc^ immunolabelling, respectively. L42 was the primary antibody of choice after comparing immunolabelling results with p4 (kindly provided by Dr. Martin H. Groschup), 6H4 (Prionics), and F89 (AH Diagnostics AS). The primary antibody was diluted in a mixture of 1 part biotin (Cat. no. SP-2001, Vector Laboratories) to 6 parts TNB. In control sections, the primary antibody was exchanged with a mouse IgG_1_ isotype control (557273, BD Biosciences) diluted to the same concentration as the primary antibody.

A secondary biotinylated sheep anti-mouse antibody, diluted 1∶200 in TNB buffer, was incubated for 30 minutes and followed by the enhancement steps of the indirect TSA-kit. Peroxidase activity was detected using 3-amino-9-ethyl carbazole (AEC) (Sigma) and counterstained with haematoxylin. All washing steps were performed with PBS containing 0.05% Tween for 3×5 minutes.

### PrP^Sc^ and PGP double immunohistochemistry

For double labelling of PrP^Sc^ and nerve fibres, formalin fixed paraffin embedded sections were pretreated as for the PrP^Sc^ immunolabelling mentioned above. To block nonspecific binding, sections were incubated with normal goat serum diluted 1∶50 in 5% bovine serum albumin dissolved in Tris-buffered saline (BSA/TBS). Mouse monoclonal antibody L42 diluted 1∶300 and polyclonal rabbit antibody PGP9.5 (Z 5116, DakoCytomation) diluted 1∶1000 in 1% BSA/TBS were incubated together on tissue sections overnight at 4°C. The further procedure was done sequentially, finishing the PrP^Sc^ immunolabelling first. The sections were incubated with secondary antibody from the EnVision+ anti-mouse HRP kit (K4005, DakoCytomation) and the peroxidase activity was detected using DAB from the same EnVision kit. The sections were then incubated with secondary antibody from the PowerVision anti-rabbit AP (DPVR-15FR-IV, AH diagnostics) and the alkaline phosphatase reactivity was detected using FastRed tablets from the same kit. Sections were counterstained with haematoxylin. All washing steps before the alkaline phosphatase conjugated secondary antibody were performed using PBS containing 0.05% Tween, and the subsequent washing steps were done with TBS without Tween. For PrP^Sc^ labelling in the double immunolabelling protocol, the EnVision protocol was preferred to the longer TSA method. A single immunohistochemical protocol for PGP9.5 labelling of nerves was tested on spleen sections before incorporating the procedure in the double immunolabelling protocol.

### In situ hybridisation

Frozen sections (12 µm) were cut with a cryostat (Leitz Cryostat 1720) and mounted on positively charged slides (Superfrost Plus; Menzel-Gläser). To increase the sensitivity, a cocktail of two digoxigenin (DIG)-labelled cRNA nucleotide fragments of the coding and the 3′-untranslated regions of *PRNP* mRNA was utilised. Each nucleotide fragment covered approximately 700 bp of the ORF or the 3′ UTR. The synthesis of the digoxigenin (DIG) labelled probes and ISH was carried out according to the method described by Austbø *et al*. (2006). For each of the analysed tissue sections, sense probes were applied to serial sections as a negative control to confirm the specificity of the hybridisation.

### Statistics

Statistical differences of mRNA expression between the spleen compartments were evaluated using the Tukey-Kramer Multiple Comparisons Test. The distribution of data sets was tested using the Kolmogorov-Smirnov normality test. Differences in expression between compartments were considered to be significant with values of probability P<0.05.

## Results

### PrP mRNA detection and quantification in tissue compartments of the spleen

**Figure 1:**
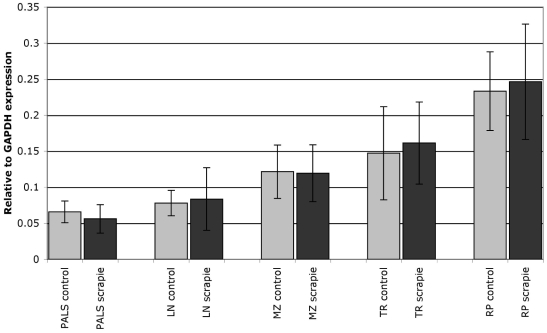
PrP mRNA expression in splenic compartments. PrP mRNA expression levels in compartments of spleen of five VRQ/VRQ lambs after oral inoculation with scrapie (black columns) and five control lambs (gray columns). The y axis represents mRNA levels relative to GAPDH expression obtained by real-time RT-PCR analysis. Error bars represent standard deviation. There was no significant difference in PrP mRNA levels between the scrapie-inoculated and control animals. The level of PrP mRNA expression in the red pulp (RP) was significantly higher than in all other tissue compartments, namely the periarterial lymphatic sheaths (PALS), lymphoid nodules (LN), marginal zone (MZ) and trabeculae (TR).

PrP mRNA expression was detected in all the microdissected tissue compartments of all animals examined. There was no significant difference in PrP mRNA levels between the scrapie-infected (n = 5) and control (n = 5) animals in any of the compartments (P>0.05; Figure 1). For the comparison of PrP mRNA expression between tissue compartments, data from the control animals were used (n = 5). A pairwise comparison of tissue compartments revealed that the level of PrP mRNA expression in the red pulp was significantly higher than the levels of expression in all other tissue compartments (P<0.05). There was no significant difference in the levels of PrP mRNA expression between the other compartments, namely lymphoid nodule, marginal zone, PALS and trabeculae.

### PrP^C^ immunohistochemistry

**Figure 2:**
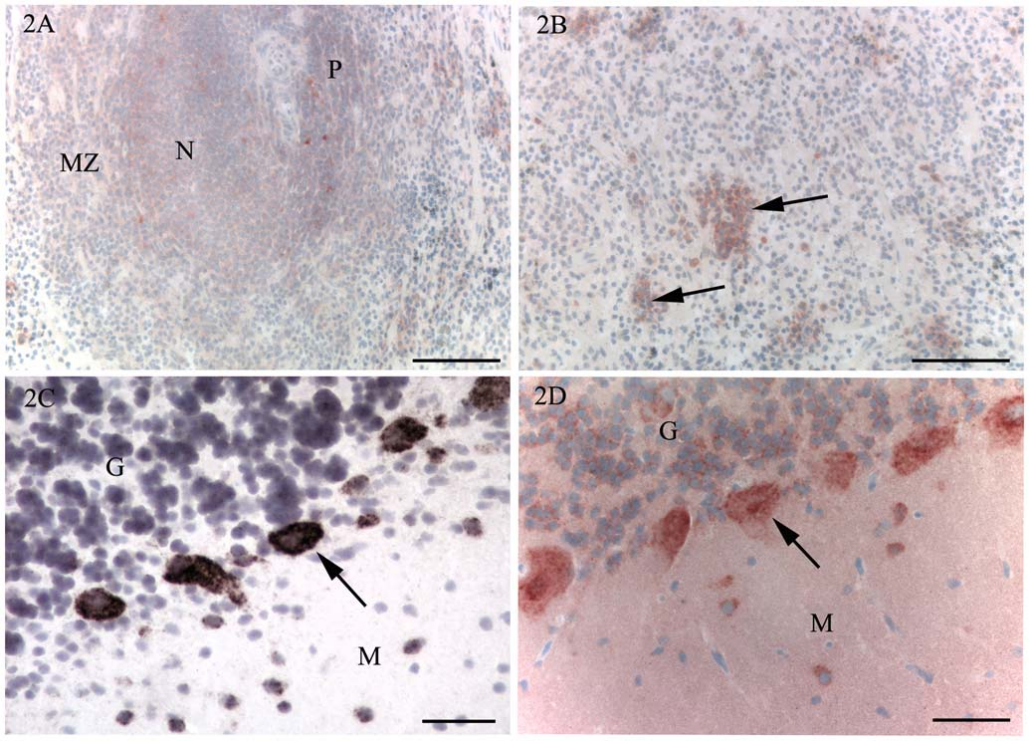
Immunohistochemistry for PrP^C^ (A, B, D) and *in situ* hybridisation for PrP mRNA (C). A. Spleen of control animal. A weak immunolabelling is present in a lymphoid nodule (N), the marginal zone (MZ) and PALS (P). B. Spleen of control animal. In the red pulp, immunolabelling for PrP^C^ was mainly detected in single cells or small groups of cells (arrows). C, D. To correlate detection of PrP^C^ and PrP mRNA, cerebellum of a normal one month old lamb was examined using immunohistochemistry and *in situ* hybridisation. C. *In situ* hybridisation. PrP transcripts were detected in Purkinje cells (arrow) and some scattered cells of the molecular (M) and granule cell (G) layers. D. PrP^C^ immunohistochemistry. There is strong cytoplasmic immunolabelling of Purkinje cells (arrows) and scattered cells of the molecular (M) and granule cell (G) layers. Note the weak diffuse immunolabelling of the molecular layer that is not present with *in situ* hybridisation (Fig. 2C). Bars, 100 µm (A, B), 50 µm (C, D). Nuclei were stained with haematoxylin.

In the spleen, a weak and diffuse immunolabelling for PrP^C^ was detected in the lymphoid nodules (Figure 2A), and a similar, but even weaker immunolabelling was present in the PALS and the marginal zone. In the scrapie inoculated animals, a few large immunolabelled cells were observed in the germinal centres, which were interpreted to be TBMs due to the presence of a similar labelling pattern with the PrP^Sc^ detection protocol (see below). In the red pulp, a weak diffuse immunolabelling was also detected. In addition, distinct cytoplasmatic immunolabelling in large mononuclear cells was detected in scattered single cells or small groups of cells (Figure 2B). Cytoplasmatic immunolabelling was also detected in monocytes in blood vessels.

Large nerve bundles were present in the spleen sections from 7 animals and weak PrP^C^ immunolabelling of nerves was detected in the spleen of 5 animals (from 3 inoculated sheep and 2 control sheep in frozen spleen specimens, not shown). No immunolabelling of Schwann cell bodies could be detected. Except for the presumed labelling of TBMs in scrapie inoculated animals (as described above), no differences in PrP^C^ immunolabelling were detected between inoculated animals and controls or between the animals euthanised at 10, 14 or 18 months post inoculation. The capsule, vessel walls, large trabeculae and bundles of smooth muscle cells in the red pulp did not show labelling for PrP^C^.

To compare the localisation of PrP^C^ with PrP mRNA expression, an ISH protocol was used on tissue sections from the spleens of scrapie-inoculated and control sheep. The ISH protocol did not reveal any specific labelling for PrP mRNA (not shown). Extended incubation with the chromogen resulted in a low level of background labelling with both the sense and antisense (negative control) probes. Protocols incorporating reduced levels of stringency produced labelling with both probes.

As a positive control for the selected ISH protocol and to allow comparison with PrP^C^ immunohistochemistry, the ISH protocol and the PrP^C^ immunolabelling protocol were performed on tissue sections from the cerebellum of a normal one month old lamb. The ISH protocol produced strong labelling of Purkinje cells and some scattered cells of the molecular and granule cell layers of the cerebellum (Figure 2C). The PrP^C^ immunolabelling protocol revealed a strong, granular, cytoplasmatic immunolabelling of Purkinje cells (Figure 2D) and neuronal immunolabelling in the granule cell layer of the cerebellum. In the molecular layer, a weak diffuse immunolabelling was observed, but there was also a stronger and granular cytoplasmic labelling of molecular layer cells. The white matter of the cerebellum did not show immunolabelling for PrP^C^. Except for the weak diffuse labelling of the molecular layer, there was a good correlation between PrP^C^ detected by immunohistochemistry and PrP mRNA detected by ISH in tissues from the cerebellum.

### PrP^Sc^ immunohistochemistry

**Figure 3:**
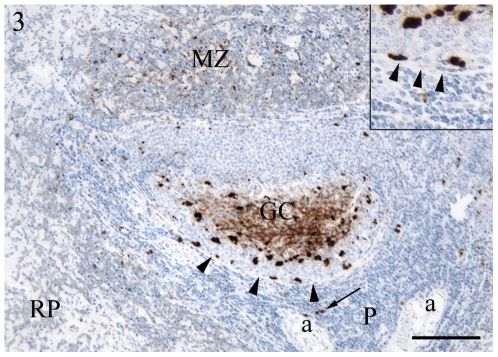
Immunohistochemistry for PrP^Sc^. Scrapie inoculated sheep. Immunolabelling was most abundant in the light zone of the germinal centre (GC) but a few scattered strongly labelled cells were also present in the dark zone near the outer rim of the lymphoid nodule delineated by spindle-shaped cells (arrowheads, also in inset). In the marginal zone (MZ), an abundant finely granular labelling was prominent and in the PALS (P) a few medium-sized granules (arrow), often close to the arteriole (a), were labelled. RP: red pulp. Bar, 100 µm. Nuclei were stained with haematoxylin.

Immunolabelling for PrP^Sc^ was detected in the spleen of all scrapie-exposed sheep and no immunolabelling was detected in any compartment of the spleen in the control animals. In the spleens of scrapie-inoculated sheep, PrP^Sc^ immunolabelling was most abundant in the lymphoid nodules and was present in two distinct labelling patterns, a fine intercellular network representing a follicular dendritic cell (FDC) pattern and as medium to large accumulations of granular material representing a TBM pattern (Figure 3). In almost all nodules, both of these PrP^Sc^ immunolabelling patterns were detected but in a few nodules only the labelling pattern typical for TBM was detected. The immunolabelling was always strongest in the light zone of germinal centres while in the dark zone of the germinal centres only scattered immunolabelled granules were detected. The outer rim of lymphoid nodules was formed by a single layer of spindle shaped reticular cells. Lymphoid nodule cells containing small to medium sized granular immunolabelled material were detected abutting this rim of reticular cells in all scrapie inoculated animals (Figure 3, inset).

In the animals showing immunolabelling for PrP^Sc^ in the marginal zone, the immunolabelling was finely granular, with granules smaller than those detected in TBMs of the nodules (Figure 3). In the PALS of all inoculated animals, only small amounts of immunolabelling were detected as medium to large sized granules localised between the lymphocytes or in close association with the central arteriole of the PALS (Figure 3). Large nerve bundles were present in sections from the spleens of 2 of 5 scrapie inoculated animals (formalin fixed spleen specimens), but no PrP^Sc^ immunolabelling was observed in any of these intrasplenic nerves. Immunolabelling was not detected in the red pulp or the large connective tissue trabeculae of the spleen of scrapie inoculated sheep.

### PGP immunolabelling of nerve fibres and association with PrP^Sc^

**Figure 4:**
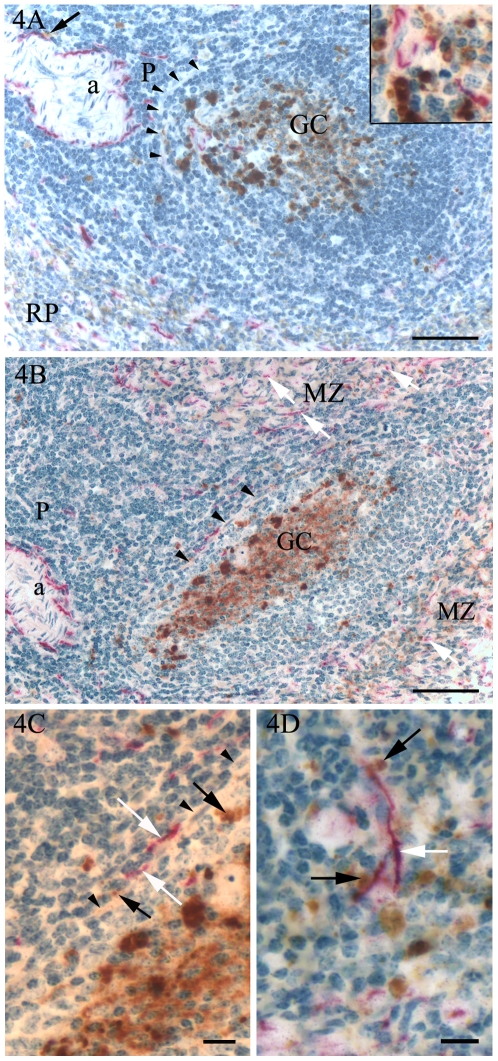
Immunohistochemistry, double labelling for PrP^Sc^ (*brown colour*) and nerves (*PGP, red colour*). Scrapie inoculated sheep. A, B. In the germinal centres (GC), a few nerve fibres were present in close association with cells strongly immunolabelled for PrP^Sc^ (A; inset). In some areas, the lymphoid nodules were separated from the PALS by a narrow rim of spindle-shaped cells (A, B; arrowheads) with PrP^Sc^ labelling mostly confined to the nodule side of the rim. In the PALS, nerves were most abundant around the arteriole (a), sometimes with PrP^Sc^-labelled granules close to the nerve fibres (A; arrow). Among the lymphoid cells of the PALS, a few nerve fibres and small granules labelled for PrP^Sc^ were generally not co-localised. In the red pulp (A; RP) and the marginal zone (B; MZ), longitudinally and cross-sectioned small nerve fibres (B; white arrows) were abundant. C. High power view of B, showing the PALS-germinal centre transition (arrowheads), PGP labelled nerve fibres (white arrows) and PrP^Sc^ (black arrows). D. In the marginal zone, the nerve fibres (white arrow) were often close to numerous small granules labelled for PrP^Sc^ (black arrows). Bars, 100 µm (A, B), 10 µm (C, D). Nuclei were stained with haematoxylin.

Double immunohistochemical labelling for PGP and PrP^Sc^ showed that in the PALS, granular PrP^Sc^ immunolabelling was detected near numerous nerve fibres immunolabelled for PGP that were found closely associated with the central arteriole. Among the lymphocytes of the PALS, PGP-labelled nerve fibres and accumulations of PrP^Sc^ were sparse and rarely showed a close association (Figure 4A, B). In lymphoid nodules with detectable nerve fibres, the few fibres present were close to the abundant PrP^Sc^ labelled cells in the light zone of the germinal centres (Figure 4A). Granular PrP^Sc^ labelling in peripheral germinal centre cells and PGP-labelled nerves of the PALS were only separated by the layer of reticular cells forming the outer rim of the nodules (Figure 4A, B, C). In the marginal zone, there were numerous thin nerve fibres generally located among smooth muscle actin-containing cells (unpublished observations) and in close association with small granules of PrP^Sc^ (Figure 4D). In the red pulp, a large number of evenly distributed thin nerve fibres showed immunolabelling for PGP, in addition to some large nerves located next to arteries, veins and lymph vessels.

## Discussion

The spleen is an organ known to accumulate substantial amounts of PrP^Sc^ during the development of scrapie in sheep [3]. It is also an organ that has been shown to support neuroinvasion in prion disease [19]. Furthermore, the level of PrP^C^ expression has been shown in rodent models to influence neuroinvasion, with high levels of PrP^C^ expression resulting in shorter incubation periods [7], [20]. The present study shows that splenic tissue compartments with significant accumulations of PrP^Sc^ had relatively low levels of PrP^C^ expression (PrP mRNA levels) while the compartment with the highest level of PrP mRNA did not support PrP^Sc^ accumulation. The process by which the prion disease agent gains entry to the nervous system has been argued to occur in two phases. Invasion of the lymphoreticular system has been distinguished from neuroinvasion proper [21]. Substantial evidence indicates that the transfer of the prion disease agent from the lymphoreticular system to the CNS occurs along peripheral nerves in a PrP^C^-dependent fashion [6], [7]. It is thus notable in the present study that the splenic lymphoid nodules and the marginal zone, which are major sites for lymphoinvasion in the spleen, as shown by extensive PrP^Sc^ accumulation, had lower levels of PrP mRNA than the red pulp, which does not accumulate PrP^Sc^. This observation supports findings in the ileal Peyer's patch of sheep where lymphoid nodules are the major sites of PrP^Sc^ accumulation in the gut wall but had low levels of PrP mRNA expression compared with other tissue compartments [8]. It would thus appear that the accumulation of PrP^Sc^ in lymphoid nodules does not depend on high compartment expression of PrP^C^.

In addition to showing that splenic lymphoid nodules express low levels of PrP^C^, the present study also showed that the lymphoid nodules possessed a sparse innervation, which questions the contribution of the large accumulation of PrP^Sc^ in splenic germinal centres to the process of neuroinvasion. Prinz *et al*. [22] suggested that the relative positioning of germinal centre FDCs and nerves in the spleen controlled the efficiency of prion neuroinvasion. These and other investigators have speculated that prions are liberated from infected FDCs and either diffuse passively (exosomes [23]) or are carried (B-cells or TBMs [24]; dendritic cells [25]) to come in contact with nerve endings in a more nerve rich tissue compartment. Subsequent studies have considered mobile cells (migratory dendritic cells, B cells or other splenic mononuclear cells) as unlikely candidates for the transfer of prions from the immune to the peripheral nervous system and instead linked efficient transmission of infection to the density of nerve fibres in the spleen, as demonstrated by the use of different knock-out mice [26]. The present study of the spleen of scrapie-infected sheep shows that there are substantial accumulations of PrP^Sc^ in tissue compartments other than lymphoid nodules, notably the marginal zone. The marginal zone is a tissue compartment surrounding the PALS and lymphoid nodules and is predominantly composed of intermediate-sized lymphocytes and various macrophage populations. The filtration of blood with the trapping of antigen and interaction of incoming cells with resident cells is an important function of the marginal zone. The marginal zone of scrapie-infected sheep contained low PrP mRNA levels but possessed numerous nerve fibres in addition to substantial amounts of PrP^Sc^. Thus, the marginal zone may contribute to the pathogenesis of scrapie in several ways, as a compartment where neuroinvasion may occur and as a source of haematogenous spread of infection. Kimberlin and Walker [19] showed that the 100-fold greater efficiency of infection by the intravenous compared with the intraperitoneal route was entirely dependent on the spleen, because splenectomy before intravenous infection reduced its efficiency to the same as that found in intraperitoneally infected non-splenectomised mice. It is not known whether this effect is due to the blood filtering capacity of the marginal zone or to the close association between nerve fibres and the walls of arteries and capillaries in the spleen. Indeed, in the present study a close association between nerve fibres and PrP^Sc^ was also observed adjacent to the central arteriole of PALS and accompanying blood vessels supplying the lymphoid nodules. A further area of interest was the outer rim of the lymphoid nodule where nerve fibres from the PALS abutted the layer of reticular cells delimiting the PrP^Sc^ laden-nodule. The significance of the outer rim of the lymphoid nodule for neuroinvasion is not known as the integrity of the rim of reticular cells needs to be established. Immuno-electron microscope investigation of this region of the sheep spleen particularly the ramification of nerve endings would provide useful information on its potential contribution to splenic neuroinvasion [27].

In mice, FDCs of lymphoid nodules are assigned a central role in the accumulation of PrP^Sc^ and expression of PrP^C^ [28]. The low levels of PrP mRNA in lymphoid nodules suggest that some of the substrate for PrP^Sc^ accumulation may not originate from the nodules but could be transported to the nodules, either in the form of PrP^C^ or smaller aggregates of PrP^Sc^. The FDCs, expressing low levels of PrP^C^ or other cells or tissues factors present in the nodules could subsequently support the conversion of PrP^C^ and/or aggregation of PrP^Sc^. Interestingly, a recent report showed that mice expressing PrP only in neuronal cells still experienced PrP^Sc^ accumulation in lymphoid nodules after challenge with scrapie [29] inviting speculation that PrP^C^ expression by FDCs is not a requirement for their PrP^Sc^ accumulation. In the present material, challenge with scrapie resulted in the expected marked accumulation of PrP^Sc^ in splenic nodules but in contrast to the findings of Austbø *et al.* [9] in ileal Peyer's patch nodules, a significant change in nodular PrP mRNA levels was not observed. This difference between lymphoid nodules in the spleen and ileal Peyer's patch of scrapie inoculated sheep may indicate the existence of alternative regulation of PrP expression between ileal and splenic nodules associated with PrP^Sc^ aggregation. Differences between Peyer's patch nodules and germinal centres of peripheral lymphatic tissues have been reported [30]. However, it cannot be excluded that the changes in protein and mRNA levels of PrP^C^ could occur in splenic compartments at time periods other than those examined in this study. It has previously been shown that accumulations of PrP^Sc^ are detectable in the spleen from approximately 10 months post inoculation in sheep of this breed with the VRQ/VRQ genotype [4].

To compare the PrP mRNA levels in the different compartments with localisation of PrP^C^, immunohistochemical studies were performed in parallel with *in situ* hybridisation (ISH). The immunohistochemical method for the detection of PrP^C^ and the ISH protocol for the localisation of PrP mRNA were established using tissue from the cerebellum of a lamb. The ISH results on cerebellar tissue were in agreement with previous reports in sheep and human [31], [32] and were in accordance with the immunohistochemical labelling obtained on the same tissue. However on splenic tissue, the ISH protocol produced no labelling and the immunolabelling for PrP^C^ was weak. A similar ISH protocol has been used to demonstrate the localisation of PrP mRNA in the ileal Peyer's patch of sheep [9] and the inability to detect expression in the spleen would suggest that the cellular levels in the spleen were below the threshold of detection of the ISH protocol. In the ileal Peyer's patch, PrP mRNA expression as detected by ISH was confined to relatively few cells of the ganglia of the enteric nervous system suggesting that the high level of expression in this particular compartment was the result of high expression in a few positive cells. In contrast, splenic PrP^C^ immunolabelling showed that many cells in the red pulp expressed PrP weakly suggesting that the relatively high PrP mRNA level in the red pulp compartment was the result of a relatively low single cell expression in a broad group of cells. The relationship between tissue compartment expression of PrP and the expression level in individual cells within that compartment could also explain the apparent inconsistency between splenic PrP^C^ immunolabelling and the quantitative PrP mRNA studies observed in the lymphoid nodules. Compared to the other compartments, the lymphoid nodules expressed low levels of PrP mRNA, but showed the strongest immunolabelling. This discrepancy may reflect the concentration of most of the lymphoid nodule PrP^C^ expression in a relatively small subpopulation of cells within the compartment, in this case presumably FDCs.

In *tga*20 mice overexpressing PrP^C^, PrP^C^ was detected immunohistochemically in Schwann cells of the sciatic nerve [33], and because the kinetics of axonal flow were incompatible with transport of scrapie infectivity, Schwann cells were proposed to have an important role in prion propagation. In the present study, immunolabelling for PrP^C^ in Schwann cells accompanying intrasplenic nerves was not detected. This lack of immunolabelling could be due to a lower level of PrP^C^ expression in wild type Schwann cells of sheep compared with that of *tga*20 mice.

In summary, our results show that the major sites of accumulation of PrP^Sc^ in the spleen of sheep experimentally infected with scrapie were in tissue compartments expressing low levels of PrP mRNA. This study thus provides further evidence that the accumulation of PrP^Sc^ is not dependent on the level of expressed PrP^C^. Further, a consideration of the density of nerves in splenic tissue compartments and the intimacy of association between PrP^Sc^ and nerves within the nerve-rich compartments of the marginal zone and PALS drew attention to the sites of possible neuro-prion contact adjacent to the wall of the central artery and of capillaries supplying germinal centres, in addition to the outer rim of germinal centres.
